# Efficacy of Pembrolizumab Monotherapy in Japanese Patients with Advanced Gastric or Gastroesophageal Junction Cancer

**DOI:** 10.1007/s12029-023-00920-9

**Published:** 2023-04-10

**Authors:** Kei Muro, Kohei Shitara, Kensei Yamaguchi, Takaki Yoshikawa, Hironaga Satake, Hiroki Hara, Naotoshi Sugimoto, Nozomu Machida, Masahiro Goto, Hisato Kawakami, Kenji Amagai, Yasushi Omuro, Taito Esaki, Shuichi Hironaka, Tomohiro Nishina, Yoshito Komatsu, Hisahiro Matsubara, Shinichi Shiratori, Shirong Han, Taroh Satoh, Atsushi Ohtsu

**Affiliations:** 1 Department of Clinical Oncology, Aichi Cancer Center Hospital, 1-1 Kanokoden, Chikusaku, Nagoya, 464-8681 Japan; 2 Department of Gastroenterology and Gastrointestinal Oncology, National Cancer Center Hospital East, Kashiwa, Japan; 3 Department of Gastroenterological Chemotherapy, The Cancer Institute Hospital of Japanese Foundation for Cancer Research, Koto City, Japan; 4 Department of Gastric Surgery, National Cancer Center Hospital, Tokyo, Japan; 5 Department of Medical Oncology, Kobe City Medical Center General Hospital, Kobe, Japan; 6 Department of Medical Oncology, Kochi Medical School, Kochi, Japan; 7 Department of Gastroenterology, Saitama Cancer Center, Saitama, Japan; 8 Department of Medical Oncology, Osaka International Cancer Institute, Osaka, Japan; 9 Division of Gastrointestinal Oncology, Shizuoka Cancer Center, Shizuoka, Japan; 10 Cancer Chemotherapy Center, Osaka Medical College, Osaka, Japan; 11 Medical Oncology, Kindai University, Osaka, Japan; 12 Department of Gastroenterology, Ibaraki Prefectural Central Hospital, Kasama, Japan; 13 Department of Medical Oncology, Tokyo Metropolitan Cancer and Infectious Disease Center Komagome Hospital, Bunkyo City, Japan; 14 Department of Gastrointestinal and Medical Oncology, National Hospital Organization Kyushu Cancer Center, Fukuoka, Japan; 15 Clinical Trial Promotion Department, Chiba Cancer Center, Chiba, Japan; 16 Clinical Oncology, National Hospital Organization Shikoku Cancer Center, Matsuyama, Japan; 17 Department of Cancer Chemotherapy, Division of Cancer Center, Hokkaido University Hospital, Sapporo, Japan; 18 Department of Frontier Surgery, Graduate School of Medicine, Chiba University, Chiba, Japan; 19 Oncology Clinical Development, MSD K.K, Tokyo, Japan; 20 Department of Frontier Science for Cancer and Chemotherapy, Osaka University Hospital, Suita, Osaka Japan; 21 Department of Gastrointestinal Oncology, National Cancer Center Hospital East, Kashiwa, Japan

**Keywords:** Biomarkers, Clinical trial, Gastrointestinal neoplasm, Pembrolizumab, Japan

## Abstract

**Purpose:**

Pembrolizumab demonstrated antitumor activity in programmed death ligand 1 positive (combined positive score (CPS) ≥ 1) gastric/gastroesophageal junction cancer in KEYNOTE-059 (third line or beyond), KEYNOTE-061 (second line), and KEYNOTE-062 (first line). We characterized efficacy and safety of pembrolizumab monotherapy in Japanese patients across several lines of therapy in these studies.

**Methods:**

This analysis was conducted in 34 patients from KEYNOTE-059 cohort 1 (all pembrolizumab), including 13 patients with CPS ≥ 1, 65 patients with CPS ≥ 1 from KEYNOTE-061 (pembrolizumab, *n* = 27; chemotherapy, *n* = 38), and 70 patients with CPS ≥ 1 from KEYNOTE-062 (pembrolizumab, *n* = 38; chemotherapy, *n* = 32). Overall survival (OS), progression-free survival (PFS), objective response rate (ORR), and safety were evaluated.

**Results:**

In KEYNOTE-059, ORR with pembrolizumab was 9%, median PFS was 2 months, and median OS was 10 months. In KEYNOTE-061, median OS was 12 months with pembrolizumab versus 10 months with chemotherapy (hazard ratio (HR), 0.67; 95% confidence interval (CI), 0.39–1.15). Median PFS (pembrolizumab vs. chemotherapy) was 2 months versus 4 months (HR, 1.21; 95% CI, 0.69–2.13); ORR was 7% versus 18%. In KEYNOTE-062, median OS was 20 months with pembrolizumab versus 18 months with chemotherapy (HR, 0.76; 95% CI, 0.43–1.33). Median PFS (pembrolizumab vs. chemotherapy) was 6 months versus 7 months (HR, 1.03; 95% CI, 0.61–1.74); ORR was 29% versus 34%.

**Conclusions:**

The current analysis provides valuable information that anti–PD-1 therapies are worthy of further assessment for gastric cancer.

**Trial Registration:**

ClinicalTrials.gov: NCT02335411 (KEYNOTE-059), NCT02370498 (KEYNOTE-061), and NCT02494583 (KEYNOTE-062).

**Supplementary Information:**

The online version contains supplementary material available at 10.1007/s12029-023-00920-9.

## Introduction

Gastric cancer (GC) is the fifth most common cancer worldwide, and the geographic distribution of GC incidence and mortality is disproportionate, with the highest rates observed in east Asian countries, such as South Korea, Mongolia, and Japan [1, 2].

In Japan, the current standard of care for unresectable advanced, recurrent, or metastatic GC in the first-line setting is systemic chemotherapy with a fluoropyrimidine plus platinum-based agent with or without nivolumab for human epidermal growth factor receptor 2 (HER2)–negative tumors or a trastuzumab-containing regimen for HER2-positive tumors [3]. Recommendations for subsequent lines of therapy include paclitaxel plus ramucirumab in the second-line setting and irinotecan or the anti–programmed death 1 (PD-1) monoclonal antibody nivolumab in the third-line setting [3].

The PD-1 inhibitor pembrolizumab was approved by the US Food and Drug Administration—based on the KEYNOTE-059 study—for recurrent locally advanced or metastatic programmed death ligand 1 (PD-L1)–positive (combined positive score (CPS) ≥ 1) gastric/gastroesophageal junction (GEJ) adenocarcinoma with disease progression on or after ≥ 2 previous lines of therapy, including fluoropyrimidine- and platinum-containing chemotherapy, and, if appropriate, HER2/neu-targeted therapy [4]. In Japan, pembrolizumab is approved for the treatment of advanced/recurrent microsatellite instability-high (MSI-H) solid tumors, including GC [5], that progressed after chemotherapy [6].

In the single-arm, multicohort, phase 2 KEYNOTE-059 study, patients with advanced gastric/GEJ cancer in cohort 1 received pembrolizumab in the third-line or later setting [7]. Among 148 patients with PD-L1-positive tumors, durable antitumor activity was observed (objective response rate (ORR), 15.5%; median duration of response (DOR), 16.3 months) with a manageable safety profile [7].

KEYNOTE-061 was a randomized, phase 3 study of second-line therapy with pembrolizumab versus paclitaxel in advanced gastric/GEJ cancer [8]. In the primary analysis (395 patients with CPS ≥ 1), pembrolizumab did not significantly improve overall survival (OS) versus paclitaxel (hazard ratio (HR), 0.82; 95% confidence interval (CI), 0.66–1.03; one-sided *P* = 0.042) [8]; post hoc analysis of patients with CPS ≥ 10 revealed a greater survival benefit with pembrolizumab than paclitaxel (HR, 0.64; 95% CI, 0.41–1.02) [9]. Similar ORRs were reported for pembrolizumab and paclitaxel (15.8% and 13.6%, respectively) in patients with CPS ≥ 1, but median DOR was longer in the pembrolizumab group (18.0 vs. 5.2 months) [8]. Pembrolizumab did not prolong progression-free survival (PFS), but its safety profile when compared with that of paclitaxel was favorable [8].

First-line therapy with pembrolizumab with or without chemotherapy (cisplatin plus 5-fluorouracil or capecitabine) versus chemotherapy was assessed in the randomized, phase 3 KEYNOTE-062 study in patients with advanced/metastatic gastric/GEJ cancer [10]. In 506 patients with CPS ≥ 1, noninferiority of pembrolizumab monotherapy versus chemotherapy for OS was met, with an HR of 0.91 (99.2% CI, 0.69–1.18; prespecified noninferiority margin, 1.2); a lower ORR (15% vs. 37%) was also observed [10]. Predefined analysis of patients with CPS ≥ 10 revealed that OS was numerically prolonged with pembrolizumab monotherapy versus chemotherapy (HR, 0.69; 95% CI, 0.49–0.97), but this difference was not statistically tested. Pembrolizumab also demonstrated better tolerability than chemotherapy.

We retrospectively evaluated Japanese patients with gastric/GEJ cancer enrolled in KEYNOTE-059, KEYNOTE-061, and KEYNOTE-062 to characterize treatment response with pembrolizumab monotherapy across several lines of therapies.

## Materials and Methods

### Study Designs

Detailed descriptions of the study designs for KEYNOTE-059 cohort 1, KEYNOTE-061, and KEYNOTE-062 have been published [7, 8, 10]; additional details appear in Online Resource 1. The current analysis focused on the subgroup of patients enrolled at Japanese sites in each study.

The study protocols and all amendments were approved by the institutional review board or ethics committee at each institution. The studies were conducted in accordance with the protocol and its amendments, Good Clinical Practice guidelines, and the Declaration of Helsinki. All patients provided written informed consent.

### Outcomes

OS, PFS, ORR, safety, and tolerability were evaluated in the pembrolizumab monotherapy and chemotherapy treatment groups.

### Statistical Analyses

Efficacy data were reported for the subgroup of Japanese patients from KEYNOTE-059 cohort 1 and the subgroup of Japanese patients with CPS ≥ 1 and CPS ≥ 10 from both KEYNOTE-061 and KEYNOTE-062 who had received ≥ 1 dose of study drug. Safety data were reported for all Japanese patients from each study. Patients could have had more than 1 immune-mediated adverse events (imAEs). Results were analyzed for each of the trials separately (i.e., results were not pooled across trials). The primary efficacy analyses were performed in the CPS ≥ 1 populations of the KEYNOTE-059 (cohort 1) and in the CPS ≥ 1 and CPS ≥ 10 populations of the KEYNOTE-061 and KEYNOTE-062 studies.

The database cutoff dates for this analysis were August 8, 2018 (KEYNOTE-059; NCT02335411); October 26, 2017 (KEYNOTE-061; NCT02370498); and March 26, 2019 (KEYNOTE-062; NCT02494583).

Additional details can be found in Online Resource 1.

## Results

### Patients

This analysis of Japanese patients included 34 patients from KEYNOTE-059, 65 patients from KEYNOTE-061, and 70 patients from KEYNOTE-062 (Table 1). Patient demographics and baseline characteristics are summarized in Table 2.

**Table 1:** Disposition of Japanese patients from the KEYNOTE-059, KEYNOTE-061, and KEYNOTE-062 studies

*n* (%)	KEYNOTE-059	KEYNOTE-061		KEYNOTE-062	
	All patients	CPS ≥ 1		CPS ≥ 1	
	Pembrolizumab	Pembrolizumab	Chemotherapy	Pembrolizumab	Chemotherapy
	*n* = 34	*n* = 27	*n* = 38	*n* = 38	*n* = 32
Status for trial					
Discontinued	31 (91)	21 (78)	35 (92)	24 (63)	25 (78)
Death	31 (91)	21 (78)	34 (90)	24 (63)	25 (78)
Protocol violation	0	0	1 (3)	0	0
Ongoing	3 (9)	6 (22)	3 (8)	14 (37)	7 (22)
Status for study treatment					
Discontinued		25 (93)	36 (100)	36 (95)	31 (97)
AE	—	0	1 (3)	3 (8)	2 (6)
Clinical progression	—	3 (11)	3 (8)	6 (16)	1 (3)
Progressive disease	—	22 (82)	32 (89)	25 (66)	25 (78)
Noncompliance	—	0	0	1 (3)	1 (3)
Withdrawal by patient	—	0	0	1 (3)	2 (6)
Ongoing	—	1 (4)	0	1 (3)	1 (3)

*AE* adverse events, *CPS* combined positive score

**Table 2:** Baseline characteristics of Japanese patients from the KEYNOTE-059, KEYNOTE-061, and KEYNOTE-062 studies

Characteristic	KEYNOTE-059	KEYNOTE-061		KEYNOTE-062	
	All patients	CPS ≥ 1		CPS ≥ 1	
	Pembrolizumab	Pembrolizumab	Chemotherapy	Pembrolizumab	Chemotherapy
	*n* = 34	*n* = 27	*n* = 38	*n* = 38	*n* = 32
Median age, years (range)	62 (39–83)	68 (38–75)	66 (27–77)	67 (28–83)	68 (44–85)
Male, *n* (%)	24 (71)	20 (74)	25 (66)	30 (79)	24 (75)
ECOG performance status, *n* (%)					
0	22 (65)	21 (78)	24 (63)	30 (79)	24 (75)
1	12 (35)	6 (22)	14 (37)	8 (21)	8 (25)
Location of primary tumor, *n* (%)					
Gastric	—	23 (85)	34 (89)	28 (74)	29 (91)
GEJ	—	4 (15)	4 (11)	10 (26)	3 (9)
No. of metastases					
0–2	—	27 (57)	28 (53)	24 (63)	27 (84)
≥ 3	—	20 (43)	25 (47)	12 (32)	24 (13)
Missing	—	0	0	2 (5)	1 (3)
No. of previous therapies for metastatic disease, *n* (%)					
2	11 (32)	—	—	—	—
3	9 (27)	—	—	—	—
4	11 (32)	—	—	—	—
≥ 5	3 (9)	—	—	—	—
Previous gastrectomy, *n* (%)	17 (50)	6 (22)	10 (26)	11 (29)	4 (13)
Histology, *n* (%)					
Tubular adenocarcinoma	30 (88)	5 (19)	8 (21)	—	—
Signet-ring cell carcinoma	2 (6)	2 (7)	1 (3)	—	—
Mixed carcinoma	2 (6)	0	0	—	—
Adenocarcinoma	0	16 (59)	26 (68)	—	—
Poorly cohesive carcinoma	0	4 (15)	3 (8)	—	—
Histological subtype, *n* (%)					
Diffuse	—	14 (52)	16 (42)	19 (50)	14 (44)
Intestinal	—	11 (41)	20 (53)	13 (34)	14 (44)
Mixed	—	1 (4)	1 (3)	3 (8)	4 (13)
Unknown	—	1 (4)	1 (3)	3 (8)	0
HER2-positive status, *n* (%)	12 (35)	4 (15)	9 (24)	0	0
MSI-H status, *n* (%)	1 (3)	2 (7)	2 (5)	3 (8)	1 (3)

*CPS* combined positive score, *ECOG* Eastern Cooperative Oncology Group, *GEJ* gastroesophageal junction, *HER2* human epidermal growth factor receptor 2, *MSI-H* microsatellite instability-high

Characteristics of patients with CPS ≥ 1 were generally similar between treatment groups in KEYNOTE-061 except for Eastern Cooperative Oncology Group performance status 1 (pembrolizumab, 22%; chemotherapy, 37%). Characteristics of patients with CPS ≥ 1 were generally similar between treatment groups in KEYNOTE-062 except for primary tumor location (GC—pembrolizumab, 74%; chemotherapy, 91%; GEJ cancer—pembrolizumab, 26%; chemotherapy, 9%) and previous gastrectomy (pembrolizumab, 29%; chemotherapy, 13%). In KEYNOTE-061, 82% of patients in the pembrolizumab group and 97% of patients in the chemotherapy group received subsequent therapy (Table S1). In KEYNOTE-062, 87% of patients in the pembrolizumab group and 84% of patients in the chemotherapy group received subsequent therapy (Table S2).

### Overall Survival

**Fig. 1:**
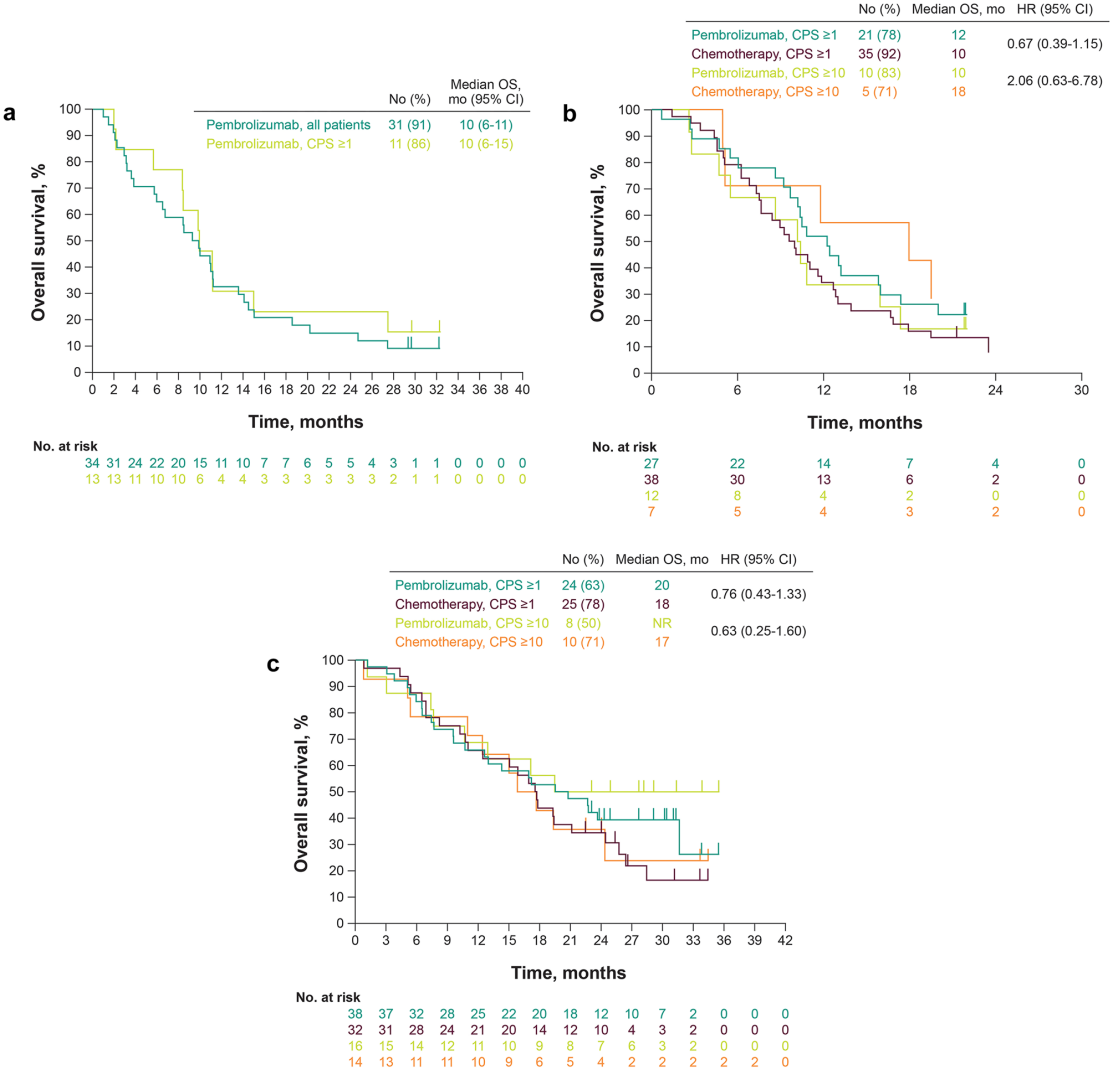
Kaplan–Meier estimates of overall survival in Japanese patients from **a** KEYNOTE-059 cohort 1, **b** KEYNOTE-061, and **c** KEYNOTE-062. *CPS* combined positive score

In KEYNOTE-059, median OS was 10 months in all patients and in the CPS ≥ 1 population (Fig. 1a).

In the CPS ≥ 1 population of KEYNOTE-061, median OS (pembrolizumab vs. chemotherapy) was 12 versus 10 months (HR, 0.67; 95% CI, 0.39–1.15); after adjusting for selected baseline characteristics in a multivariate analysis, the HR was 0.71 (95% CI, 0.40–1.25). In the CPS ≥ 10 population, median OS (pembrolizumab vs. chemotherapy) was 10 versus 18 months (HR, 2.06; 95% CI, 0.63–6.78) (Fig. 1b).

In the CPS ≥ 1 population of KEYNOTE-062, median OS (pembrolizumab vs. chemotherapy) was 20 versus 18 months (HR, 0.76; 95% CI, 0.43–1.33) (Fig. 1c); after adjusting for selected baseline characteristics in a multivariate analysis, the HR was 0.58 (95% CI, 0.30–1.10). In the CPS ≥ 10 population, median OS (pembrolizumab vs. chemotherapy) was not reached (NR) versus 17 months (HR, 0.63; 95% CI, 0.25–1.60) (Fig. 1c); after adjusting for selected baseline characteristics in a multivariate analysis, the HR was 0.49 (95% CI, 0.17–1.37).

### Progression-Free Survival

**Fig. 2:**
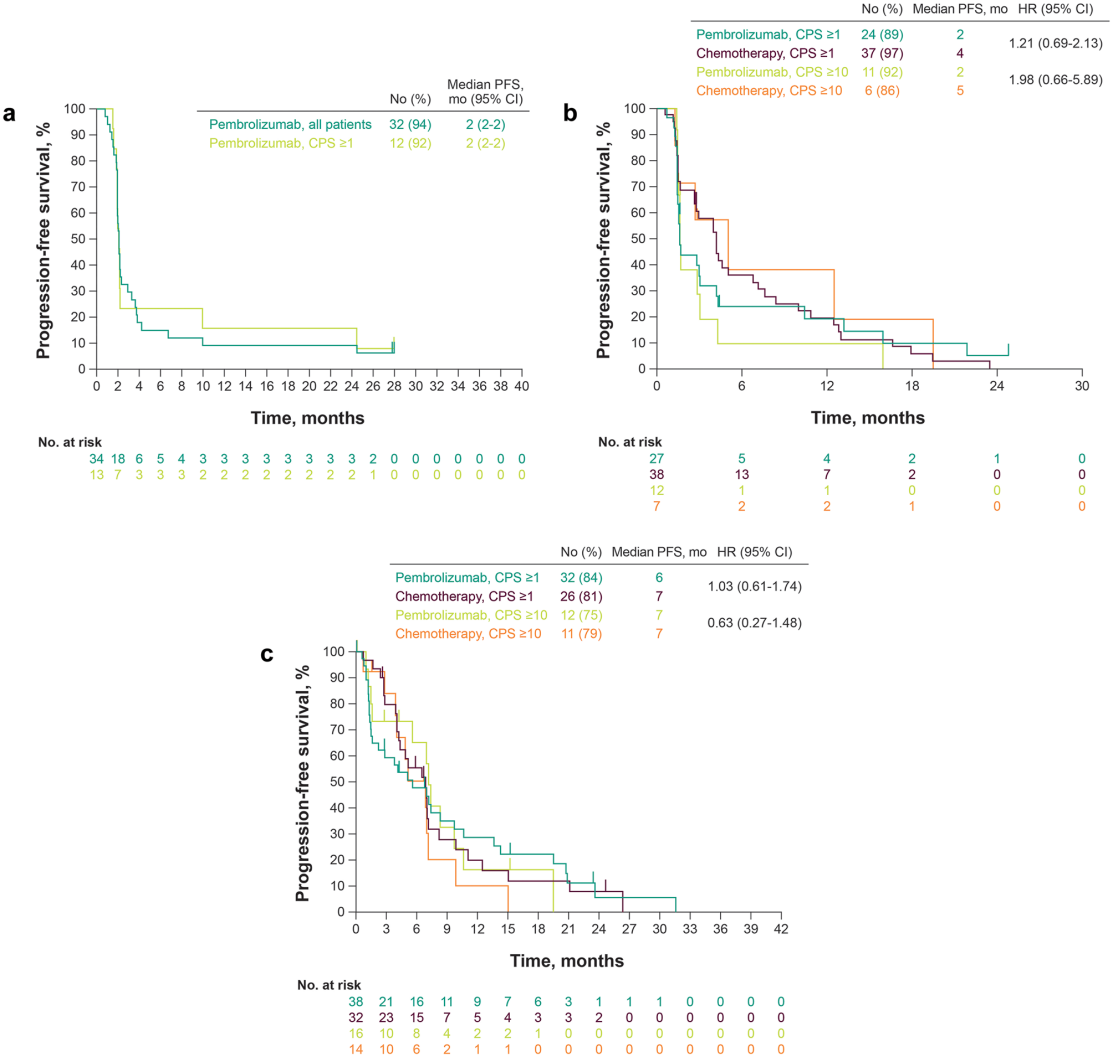
Kaplan–Meier estimates of progression-free survival in Japanese patients from **a** KEYNOTE-059 cohort 1, **b** KEYNOTE-061, and **c** KEYNOTE-062. *CPS* combined positive score

In KEYNOTE-059, median PFS based on independent central review was 2 months in all patients and in the CPS ≥ 1 population (Fig. 2a).

In the CPS ≥ 1 population of KEYNOTE-061, median PFS (pembrolizumab vs. chemotherapy) was 2 versus 4 months (HR, 1.21; 95% CI, 0.69–2.13) (Fig. 2b); after adjusting for selected baseline characteristics in a multivariate analysis, the HR was 1.22 (95% CI, 0.69–2.14). In the CPS ≥ 10 population, median PFS (pembrolizumab vs. chemotherapy) was 2 versus 5 months (HR, 1.98; 95% CI, 0.66–5.89).

In the CPS ≥ 1 population of KEYNOTE-062, median PFS (pembrolizumab vs. chemotherapy) was 6 versus 7 months (HR, 1.03; 95% CI, 0.61–1.74) (Fig. 2c); after adjusting for selected baseline characteristics in a multivariate analysis, the HR was 0.87 (95% CI, 0.49–1.55). In the CPS ≥ 10 population, median PFS (pembrolizumab vs. chemotherapy) was 7 months in each group (HR, 0.63; 95% CI, 0.27–1.48) (Fig. 2c); after adjusting for selected baseline characteristics in a multivariate analysis, the HR was 0.54 (95% CI, 0.21–1.41).

### Response

In KEYNOTE-059, ORR was 9% (*n* = 3) (Table 3). In the CPS ≥ 1 population, ORR was 15% (*n* = 2). Among the three patients in the CPS ≥ 10 population, one patient achieved an objective response (partial response (PR)).

**Table 3:** Summary of treatment response in Japanese patients from the KEYNOTE-059, KEYNOTE-061, and KEYNOTE-062 studies

*n* (%)	KEYNOTE-059	KEYNOTE-059	KEYNOTE-061		KEYNOTE-061		KEYNOTE-062		KEYNOTE-062	
	All patients	CPS ≥ 1	CPS ≥ 1		CPS ≥ 10		CPS ≥ 1		CPS ≥ 10	
	Pembrolizumab	Pembrolizumab	Pembrolizumab	Chemotherapy	Pembrolizumab	Chemotherapy	Pembrolizumab	Chemotherapy	Pembrolizumab	Chemotherapy
	*n* = 34	*n* = 13	*n* = 27	*n* = 38	*n* = 12	*n* = 7	*n* = 38	*n* = 32	*n* = 16	*n* = 14
ORR	3 (9)	2 (15)	2 (7)	7 (18)	0	1 (14)	11 (29)	11 (34)	5 (31)	4 (29)
CR	2 (6)	1 (8)	0	1 (3)	0	0	3 (8)	2 (6)	2 (13)	0
PR	1 (3)	1 (8)	2 (7)	6 (16)	0	1 (14)	8 (21)	9 (28)	3 (19)	4 (29)
SD	6 (18)	1 (8)	10 (37)	16 (42)	5 (42)	4 (57)	9 (24)	13 (41)	5 (31)	7 (50)
PD	23 (68)	10 (77)	14 (52)	12 (32)	7 (58)	2 (29)	4 (11)	2 (6)	3 (19)	1 (7)
Not available^a^	2 (6)	0	1 (4)	3 (8)	0	0	5 (13)	4 (13)	3 (19)	2 (14)

*CPS* combined positive score, *CR* complete response, *ORR* objective response rate, *PD* progressive disease, *PR* partial response, *SD* stable disease

^a^Not available includes patients who were not evaluable and patients with no postbaseline assessment as of the data cutoff date

In the CPS ≥ 1 population of KEYNOTE-061, ORR (pembrolizumab vs. chemotherapy) was 7% versus 18% (Table 3). Among the four patients with MSI-H, one chemotherapy-treated patient achieved an objective response (PR); no pembrolizumab-treated patients achieved an objective response. In the CPS ≥ 10 population, one chemotherapy-treated patient achieved objective response (PR); no pembrolizumab-treated patients achieved objective response.

In the CPS ≥ 1 population of KEYNOTE-062, ORR (pembrolizumab vs. chemotherapy) was 29% versus 34% (Table 3). Among the four patients with MSI-H, one pembrolizumab-treated patient achieved an objective response (PR); no chemotherapy-treated patients achieved objective response. In the CPS ≥ 10 population, ORR (pembrolizumab vs. chemotherapy) was 31% versus 29% (Table 3).

### Safety and Tolerability

Table 4 summarizes AEs reported in the Japanese subgroups of KEYNOTE-059, KEYNOTE-061, and KEYNOTE-062. In KEYNOTE-059, 59% of patients had ≥ 1 treatment-related AE (TRAE); 18% experienced a grade 3 or 4 event, with no grade 5 events reported. The most common any-grade TRAE (≥ 10%) was rash (Table S3). Eight patients (24%) experienced imAEs. The most common imAE was hypothyroidism (*n* = 3; 9%), followed by infusion reactions (*n* = 2; 6%), colitis, encephalitis, hyperthyroidism, pneumonitis, and severe skin reactions (*n* = 1 each; 3%).

**Table 4:** Summary of adverse events in Japanese patients from the KEYNOTE-059, KEYNOTE-061, and KEYNOTE-062 studies

*n* (%)	KEYNOTE-059	KEYNOTE-061		KEYNOTE-062	
	All patients	All patients		All patients	
	Pembrolizumab	Pembrolizumab	Chemotherapy	Pembrolizumab	Chemotherapy
	*n* = 34	*n* = 47	*n* = 50	*n* = 38	*n* = 32
≥ 1 AE	30 (88)	41 (87)	48 (96)	36 (95)	32 (100)
TRAE	20 (59)	23 (49)	46 (92)	22 (58)	30 (94)
Grade 3–5 AEs	15 (44)	14 (30)	26 (52)	14 (37)	22 (69)
Grade 3–5 TRAE	6 (18)^a^	2 (4)	22 (44)	8 (21)	19 (59)
Serious AE	9 (27)	7 (15)	4 (8)	8 (21)	11 (34)
Serious TRAE	4 (12)	0 (0)	1 (2)	5 (13)	7 (22)
Discontinuation due to AE	2 (6)	0 (0)	3 (6)	3 (8)	9 (28)
Discontinuation due to TRAE	2 (6)	0 (0)	3 (6)	2 (5)	9 (28)
Death	0 (0)	0 (0)	0 (0)	0 (0)	0 (0)

*AE* adverse event, *TRAE* treatment-related adverse event

^a^No grade 5 TRAEs occurred

In KEYNOTE-061, the incidence of any-grade TRAEs (pembrolizumab vs. chemotherapy) was 49% versus 92%; grade 3–5 events were reported in 4% versus 44% of patients (Table 4). The most common any-grade TRAEs with pembrolizumab (≥ 10%) were diarrhea, pruritus, and rash; the most common with chemotherapy (≥ 30%) were alopecia, decreased neutrophil count, and peripheral sensory neuropathy (Table S4). The incidence of imAEs (pembrolizumab vs. chemotherapy) was 19% (*n* = 9) versus 4% (*n* = 2). The most common imAE with pembrolizumab was hypothyroidism (*n* = 5, 11%), followed by infusion reactions (*n* = 2; 4%), hyperthyroidism, hypophysitis, and pneumonitis (*n* = 1 each; 2%).

In KEYNOTE-062, the incidence of any-grade TRAEs (pembrolizumab vs. chemotherapy) was 58% versus 94%; grade 3–5 events were reported in 21% versus 59% (Table 4). The most common TRAEs with pembrolizumab (≥ 10%) were pruritus, decreased appetite, diarrhea, and rash; the most common with chemotherapy (≥ 40%) were decreased appetite, nausea, decreased neutrophil count, and palmar-plantar erythrodysesthesia syndrome (Table S5). The incidence of imAEs (pembrolizumab vs. chemotherapy) was 16% (*n* = 6) versus 6% (*n* = 2). The most common imAE with pembrolizumab was colitis (*n* = 2; 5%), followed by adrenal insufficiency, hyperthyroidism, hypophysitis, hypothyroidism, myositis, and pneumonitis (*n* = 1 each; 3%).

## Discussion

These subgroup analyses of Japanese patients with gastric/GEJ cancer demonstrated that pembrolizumab monotherapy, given as first-, second-, or third-line and later therapy, demonstrated a trend toward improvement in clinical outcomes, although the results were not statistically significant compared with placebo. Data from KEYNOTE-061 and KEYNOTE-062 also demonstrated numeric improvement in median OS with pembrolizumab monotherapy compared with chemotherapy in patients with PD-L1 CPS ≥ 1. Analyses from KEYNOTE-062 also suggested improvements in median OS and PFS with pembrolizumab in patients with CPS ≥ 10; however, these differences were not statistically tested. In all three studies, pembrolizumab was generally well tolerated, produced no unexpected toxicity, and had a better safety profile than chemotherapy.

Findings from these Japanese subgroup analyses are comparable with outcomes observed in the intention-to-treat (ITT) populations of each respective study. In KEYNOTE-059, OS and PFS medians were similar between the Japanese subgroup and the ITT population [7]. Although the number of patients in the CPS ≥ 1 Japanese subgroup was small, response data were consistent with those of the ITT population with PD-L1-positive tumors [7].

Similar outcomes were also observed in the Japanese subgroup and the ITT population in KEYNOTE-061 patients with CPS ≥ 1 for OS and PFS medians, and no significant differences were observed in either population between pembrolizumab and chemotherapy [8].

In KEYNOTE-062, median OS in the Japanese subgroup with CPS ≥ 1 was higher in the pembrolizumab and chemotherapy groups (20 and 18 months, respectively) than in the ITT population (11 and 11 months), but no significant between-treatment differences were observed in either population; median PFS in the pembrolizumab group of the Japanese subgroup was also higher (6 months) than it was in the ITT population (2 months) [10]. It is notable that a greater proportion of the Japanese subgroup than the ITT population had 0 to 2 metastases (73% and 53%, respectively) and Eastern Cooperative Oncology Group performance status 0 (77% and 48%) [10].

When multiple factors influence prognosis, a combination of these factors can affect the OS HR between two treatment groups. To confirm whether the HRs were robust in our study, we performed multivariate analysis of OS after adjusting for selected baseline characteristics. We found that OS HRs ranged from 0.5 to 0.7 for an imbalanced combination of factors, supporting the robustness of our results. Furthermore, in KEYNOTE-062, 134 of 254 pembrolizumab-treated patients (53%) and 132 of 244 chemotherapy-treated patients (54%) received ≥ 1 subsequent anticancer therapy in the total population [10] compared with 33 of 38 patients (87%) and 27 of 32 (84%) patients, respectively, in the Japanese subgroup. As a result, the observed survival advantage in the Japanese subgroup may be the result of the higher proportion of patients receiving subsequent anticancer therapy. This suggests that switching to the next treatment at an appropriate time and to subsequent treatment may contribute to prolongation of survival. Further investigation of immunologic profile differences is also warranted.

PD-L1 expression can be used in clinical practice to help select patients for treatment with immunotherapy in tumor types with high PD-L1 expression. In GC, use of CPS is a robust and reproducible predictive marker to identify patients likely to respond to pembrolizumab [11]. Recent evidence suggests that enriching for PD-L1 status by increasing the minimum proportion of stained cells (e.g., from CPS ≥ 1 to CPS ≥ 10) is positively correlated with the OS benefit provided by PD-1/L1 inhibitor therapy in solid tumors [12]. In the current report, data from Japanese patients with CPS ≥ 10 from the KEYNOTE-062 study revealed an enhanced treatment effect with pembrolizumab monotherapy on survival outcomes, improving median OS (from 20 months (CPS ≥ 1) to NR (CPS ≥ 10)). Recent analyses of patients with CPS ≥ 10 from the ITT populations of KEYNOTE-059, KEYNOTE-061, and KEYNOTE-062 demonstrated improvement in OS with pembrolizumab monotherapy when a higher CPS cutoff was used [9], thus supporting the current findings in Japanese patients with CPS ≥ 10. Further enrichment of PD-L1 expression status in patients with gastric/GEJ cancer may serve as an important predictive biomarker for the selection of patients likely to benefit most from pembrolizumab monotherapy.

Pembrolizumab monotherapy was generally well tolerated in the Japanese subgroups of KEYNOTE-059, KEYNOTE-061, and KEYNOTE-062, which were comparable with the ITT population [7, 8, 10]. Most TRAEs were mild or moderate in severity, discontinuation rates were low, and the incidence of imAEs was low. The consistency of these findings suggested that the use of pembrolizumab monotherapy is generally safe across first and subsequent lines of therapy in patients with advanced/metastatic gastric/GEJ cancer.

The results of the current report for the subgroup of patients enrolled at Japanese sites in the KEYNOTE-059, KEYNOTE-061, and KEYNOTE-062 studies demonstrated the efficacy and safety of pembrolizumab in previously untreated and treated Japanese patients and showed general consistency with data from globally conducted studies. These results not only build on existing data from global studies but add to the body of evidence evaluating pembrolizumab in various patient populations in the gastric cancer treatment setting. Analyses that differ in geographic region of enrollment enable regulatory agencies and health care providers to make decisions based on the benefit-to-risk ratio of pembrolizumab across various patient populations and ultimately facilitate swift access to needed treatments by patients, which is especially important in the gastric cancer disease setting.

Limitations of the current retrospective analysis include small sample sizes for the Japanese subgroups in each study (≤ 100 patients) as well subgroups for CPS ≥ 10 and MSI-H status; thus, results should be interpreted with caution. Additionally, the current analysis included the KEYNOTE-061 study, which did not meet its primary end points of OS and PFS in patients with a PD-L1 CPS ≥ 1.

## Conclusions

These data in Japanese patients indicate that pembrolizumab monotherapy provides consistent survival benefit and an acceptable safety profile when used in the first-line (KEYNOTE-062), second-line (KEYNOTE-061), or third-line or later (KEYNOTE-059) settings in patients with advanced/metastatic gastric/GEJ cancers. The current analysis provides valuable insight and information that anti–PD-1 therapies are worthy of further assessment, particularly in patients with locally advanced/unresectable or metastatic gastric cancer. Furthermore, PD-L1 CPS ≥ 1 can be used as a predictive biomarker of response to pembrolizumab monotherapy; increasing this cutoff to CPS ≥ 10 has the potential to improve patient selection. Adequately powered, prospective clinical trials are needed to validate the optimal use of CPS as a predictive biomarker for patients with gastric/GEJ cancer.

### Supplementary Information

Below is the link to the electronic supplementary material.

Supplementary file1 (DOCX 35 KB)

## Data Availability

Merck Sharp & Dohme LLC, a subsidiary of Merck & Co., Inc., Rahway, NJ, USA (MSD), is committed to providing qualified scientific researchers access to anonymized data and clinical study reports from the company’s clinical trials for the purpose of conducting legitimate scientific research. MSD is also obligated to protect the rights and privacy of trial participants and, as such, has a procedure in place for evaluating and fulfilling requests for sharing company clinical trial data with qualified external scientific researchers. The MSD data sharing website (available at: http://engagezone.msd.com/ds_documentation.php) outlines the process and requirements for submitting a data request. Applications will be promptly assessed for completeness and policy compliance. Feasible requests will be reviewed by a committee of MSD subject matter experts to assess the scientific validity of the request and the qualifications of the requestors. In line with data privacy legislation, submitters of approved requests must enter into a standard data-sharing agreement with MSD before data access is granted. Data will be made available for request after product approval in the US and EU or after product development is discontinued. There are circumstances that may prevent MSD from sharing requested data, including country or region-specific regulations. If the request is declined, it will be communicated to the investigator. Access to genetic or exploratory biomarker data requires a detailed, hypothesis-driven statistical analysis plan that is collaboratively developed by the requestor and MSD subject matter experts; after approval of the statistical analysis plan and execution of a data-sharing agreement, MSD will either perform the proposed analyses and share the results with the requestor or will construct biomarker covariates and add them to a file with clinical data that is uploaded to an analysis portal so that the requestor can perform the proposed analyses.

## Declarations

### Disclaimer

The study funder participated in study design, data interpretation, and the writing of this report. The sponsor maintained the study database.
