# Supplementary material for: Efficacy of Pembrolizumab Monotherapy in Japanese Patients with Advanced Gastric or Gastroesophageal Junction Cancer
Source: J Gastrointest Cancer. 2023 Apr 10;54(3):951–61. doi: 10.1007/s12029-023-00920-9 (PMC10613141; doi:10.1007/s12029-023-00920-9)
Supplement: Supplementary file 1 — Supplementary file1 (DOCX 35 KB) [file 12029_2023_920_MOESM1_ESM.docx]

**Supplementary Material**

*Journal of Gastrointestinal Cancer*

Efficacy of pembrolizumab monotherapy in Japanese patients with advanced gastric or gastroesophageal junction cancer

Kei Muro,^1^ Kohei Shitara,^2^ Kensei Yamaguchi,^3^ Takaki Yoshikawa,^4^ Hironaga Satake,^5^ Hiroki Hara,^6^ Naotoshi Sugimoto,^7^ Nozomu Machida,^8^ Masahiro Goto,^9^ Hisato Kawakami,^10^ Kenji Amagai,^11^ Yasushi Omuro,^12^ Taito Esaki,^13^ Shuichi Hironaka,^14^ Tomohiro Nishina,^15^ Yoshito Komatsu,^16^ Hisahiro Matsubara,^17^ Shinichi Shiratori,^18^ Shirong Han,^18^ Taroh Satoh,^19^ Atsushi Ohtsu^20^

^1^Department of Clinical Oncology, Aichi Cancer Center Hospital, Nagoya, Japan

^2^Department of Gastroenterology and Gastrointestinal Oncology, National Cancer Center Hospital East, Kashiwa, Japan

^3^Department of Gastroenterological Chemotherapy, The Cancer Institute Hospital of Japanese Foundation for Cancer Research, Koto City, Japan

^4^Department of Gastroenterology and Hepatology, National Cancer Center Hospital, Chuo City, Japan

^5^Department of Medical Oncology, Kobe City Medical Center General Hospital, Kobe, Japan and Department of Medical Oncology, Kochi Medical School, Kochi, Japan

^6^Department of Gastroenterology, Saitama Cancer Center, Saitama, Japan

^7^Department of Medical Oncology, Osaka International Cancer Institute, Osaka, Japan

^8^Division of Gastrointestinal Oncology, Shizuoka Cancer Center, Shizuoka, Japan

^9^Cancer Chemotherapy Center, Osaka Medical College, Osaka, Japan

^10^Medical Oncology, Kindai University, Osaka, Japan

^11^Department of Gastroenterology, Ibaraki Prefectural Central Hospital, Kasama, Japan

^12^Department of Medical Oncology, Tokyo Metropolitan Cancer and Infectious Disease Center Komagome Hospital, Bunkyo City, Japan

^13^Department of Gastrointestinal and Medical Oncology, National Hospital Organization Kyushu Cancer Center, Fukuoka, Japan

^14^Clinical Trial Promotion Department, Chiba Cancer Center, Chiba, Japan

^15^Clinical Oncology, National Hospital Organization Shikoku Cancer Center, Matsuyama, Japan

^16^Department of Cancer Chemotherapy, Division of Cancer Center, Hokkaido University Hospital, Sapporo, Japan

^17^Department of Frontier Surgery, Graduate School of Medicine, Chiba University, Chiba, Japan

^18^Oncology Clinical Development, MSD K.K., Chiyoda City, Japan

^19^Department of Frontier Science for Cancer and Chemotherapy, Osaka University Hospital, Suita, Osaka, Japan

^20^Department of Gastrointestinal Oncology, National Cancer Center Hospital East, Kashiwa, Japan

**Corresponding author:**

Kei Muro, MD, PhD

Aichi Cancer Center Hospital

- 1. Kanokoden, Chikusaku

Nagoya, 464-8681, Japan

Tel.: +81 52 762 6111

Fax: +81 52 764 9855

Email: kmuro@aichi-cc.jp

**Methods**

KEYNOTE-059 cohort 1, KEYNOTE-061, and KEYNOTE-062 enrolled patients with advanced gastric/gastroesophageal junction (GEJ) cancer and evaluated the efficacy and safety of pembrolizumab 200 mg administered intravenously every 3 weeks for up to 35 cycles (~2 years). In cohort 1 of KEYNOTE-059, all patients whose disease had progressed after ≥2 previous chemotherapy regimens received pembrolizumab monotherapy. In KEYNOTE-061, patients who experienced disease progression after first-line chemotherapy with a platinum and a fluoropyrimidine were randomly assigned 1:1 to receive pembrolizumab monotherapy or chemotherapy with standard-dose paclitaxel (80 mg/m² intravenously on days 1, 8, and 15 of each 4-week cycle). In KEYNOTE-062, patients were randomly assigned 1:1:1 to receive first-line pembrolizumab monotherapy, pembrolizumab plus chemotherapy (cisplatin 80 mg/m^2^ plus 5-fluorouracil 800 g/m^2^/day on days 1 to 5 every 3 weeks [or capecitabine 1000 mg/m^2^ twice daily on days 1 to 14 every 3 weeks]) or placebo plus chemotherapy.

In KEYNOTE-059, patients were enrolled regardless of programmed death ligand 1 (PD-L1) expression status. In KEYNOTE-061, patients were also initially enrolled regardless of PD-L1 expression status, but the independent data monitoring committee subsequently recommended restricting enrolment to patients with combined positive score (CPS) ≥1 tumors. In KEYNOTE-062, patients were required to have CPS ≥1 tumors.

Assessment of primary efficacy and safety outcomes has been described in detail elsewhere [1–3]. Response and disease progression were assessed per Response Evaluation Criteria in Solid Tumors, version 1.1 (RECIST v1.1), by independent central review. Adverse events (AEs) were graded according to National Cancer Institute Common Terminology Criteria for Adverse Events (version 4.0).

*PD-L1 expression and MSI status*

PD-L1 expression was assessed in archival or newly collected tumor samples using PD-L1 IHC 22C3 pharmDx (Agilent Technologies) and were measured using CPS (defined as the number of PD-L1–positive cells [tumor cells, lymphocytes, macrophages] divided by the total number of tumor cells, multiplied by 100) [4].

Microsatellite instability (MSI) status was assessed using the Promega MSI Analysis System to investigate five mononucleotide repeat markers (*NR21, NR24, BAT25, BAT26, MONO27*). Tumors were identified as MSI-H if ≥2 markers were changed compared with the normal (blood) control.

The Kaplan-Meier method was used to calculate overall survival (OS) and progression-free survival (PFS). In KEYNOTE-061 and KEYNOTE-062, treatment differences in OS and PFS were assessed using a stratified log-rank test; hazard ratios and their associated 95% confidence intervals (CIs) were calculated using stratified Cox proportional hazards models with the Efron method of handling ties. In KEYNOTE-059, ORR with 95% CI was calculated using the Clopper-Pearson method; the stratified Miettinen and Nurminen method was used in KEYNOTE-061 and KEYNOTE-062.

*Multivariate analysis*

Multivariate analysis of PFS and OS was performed for KEYNOTE-061 and KEYNOTE-062 using baseline characteristics factors with a comparative difference between the treatment groups (~10%). In KEYNOTE-061, characteristics were primary location at diagnosis (adenocarcinoma of the gastroesophageal junction vs. adenocarcinoma of the stomach), age (≤65 vs. >65 years), ECOG performance status (0 vs. 1), histologic subtype (diffuse vs. other), and HER2 status (positive vs. negative). In KEYNOTE-062, characteristics were primary location at diagnosis (adenocarcinoma of the gastroesophageal junction vs. adenocarcinoma of the stomach), age (<65 vs. ≥65 years), oncologic surgery (gastrectomy [yes vs. no]), and number of metastases (0-2 vs. ≥3).

**Table S1**

Summary of subsequent therapy in Japanese patients from KEYNOTE-061.

| **Subsequent therapy, n (%)** | **KEYNOTE-061** | |
| --- | --- | --- |
|  | **Pembrolizumab**  **n = 27** | **Chemotherapy**  **n = 36** |
| One or more subsequent therapies (>0% of either group) | 22 (82) | 35 (97) |
| One or more subsequent therapies (≥5% of either group) |  |  |
| Paclitaxel | 20 (74) | 3 (8) |
| Ramucirumab | 18 (67) | 19 (53) |
| Irinotecan hydrochloride | 10 (37) | 26 (72) |
| Gimeracil (+) oteracil potassium (+) tegafur | 3 (11) | 5 (14) |
| Oxaliplatin | 3 (11) | 10 (28) |
| Fluorouracil | 2 (7) | 4 (11) |
| Levoleucovorin calcium | 2 (7) | 4 (11) |
| Paclitaxel albumin | 1 (4) | 2 (6) |
| Capecitabine | 0 | 2 (6) |
| Nivolumab | 0 | 4 (11) |
| Unspecified | 2 (7) | 3 (8) |

**Table S2**

Summary of subsequent therapy in Japanese patients from KEYNOTE-062.

| **Subsequent therapy , n (%**) | **KEYNOTE-062** | |
| --- | --- | --- |
|  | **Pembrolizumab**  **n = 38** | **Chemotherapy**  **n = 32** |
| One or more subsequent therapies (>0% of either group) | 33 (87) | 27 (84) |
| One or more subsequent therapies (≥5% of either group) |  |  |
| Gimeracil (+) oteracil potassium (+) tegafur | 27 (71) | 4 (13) |
| Oxaliplatin | 24 (63) | 4 (13) |
| Paclitaxel | 19 (50) | 24 (75) |
| Ramucirumab | 15 (40) | 22 (69) |
| Irinotecan hydrochloride | 10 (26) | 11 (34) |
| Fluorouracil | 4 (11) | 2 (6) |
| Cisplatin | 3 (8) | 0 |
| Levoleucovorin calcium | 3 (8) | 2 (6) |
| Capecitabine | 2 (5) | 0 |
| Docetaxel | 2 (5) | 0 |
| Nivolumab | 2 (5) | 13 (41) |
| Paclitaxel albumin | 2 (5) | 3 (9) |
| Regorafenib | 0 | 2 (6) |

**Table S3**

Summary of treatment-related AEs in Japanese patients from KEYNOTE-059 cohort 1.

| Treatment-related AE (≥5%), n (%) | KEYNOTE-059 |
| --- | --- |
|  | Pembrolizumab  n = 34 |
| Rash | 7 (21) |
| Abnormal hepatic function | 3 (9) |
| Decreased appetite | 3 (9) |
| Hypothyroidism | 3 (9) |
| Stomatitis | 3 (9) |
| Arthralgia | 2 (6) |
| Dry skin | 2 (6) |
| Dysgeusia | 2 (6) |
| Malaise | 2 (6) |
| Pruritus | 2 (6) |

*AE* adverse event.

**Table S4**

Summary of treatment-related AEs in Japanese patients from KEYNOTE-061.

| Treatment-related AE (≥5% of either group), n (%) | KEYNOTE-061 | |
| --- | --- | --- |
|  | Pembrolizumab  n = 47 | Chemotherapy  n = 50 |
| Diarrhea | 6 (13) | 5 (10) |
| Pruritus | 5 (11) | 1 (2) |
| Rash | 5 (11) | 2 (4) |
| Decreased appetite | 4 (9) | 12 (24) |
| Arthralgia | 2 (4) | 7 (14) |
| Constipation | 2 (4) | 4 (8) |
| Myalgia | 2 (4) | 9 (18) |
| Nausea | 2 (4) | 8 (16) |
| Pyrexia | 2 (4) | 4 (8) |
| Stomatitis | 2 (4) | 5 (10) |
| Fatigue | 1 (2) | 8 (16) |
| Malaise | 1 (2) | 8 (16) |
| Alopecia | 0 | 36 (72) |
| Anemia | 0 | 4 (8) |
| Neutropenia | 0 | 4 (8) |
| Neutrophil count decreased | 0 | 17 (34) |
| Peripheral neuropathy | 0 | 7 (14) |
| Peripheral sensory neuropathy | 0 | 16 (32) |
| White blood cell count decreased | 0 | 14 (28) |

*AE* adverse event.

**Table S5**

Summary of treatment-related AEs in Japanese patients from KEYNOTE-062.

| Treatment-related AE (≥5% in either group), n (%) | KEYNOTE-062 | |
| --- | --- | --- |
|  | Pembrolizumab  n = 38 | Chemotherapy  n = 32 |
| Pruritus | 7 (18) | 2 (6) |
| Decreased appetite | 5 (13) | 24 (75) |
| Diarrhea | 5 (13) | 7 (22) |
| Rash | 5 (13) | 1 (3) |
| Rash maculopapular | 4 (11) | 2 (6) |
| Aspartate transaminase increased | 3 (8) | 0 |
| Fatigue | 3 (8) | 7 (22) |
| Alanine aminotransferase increased | 2 (5) | 0 |
| Anemia | 2 (5) | 6 (19) |
| Arthralgia | 2 (5) | 0 |
| Cheilitis | 2 (5) | 1 (3) |
| Dermatitis | 2 (5) | 0 |
| Hyponatremia | 2 (5) | 3 (9) |
| Vomiting | 2 (5) | 6 (19) |
| Blood creatinine increased | 1 (3) | 6 (19) |
| Constipation | 1 (3) | 9 (28) |
| Dry skin | 1 (3) | 3 (9) |
| Dysgeusia | 1 (3) | 5 (16) |
| Hypoalbuminemia | 1 (3) | 2 (6) |
| Malaise | 1 (3) | 5 (16) |
| Neutrophil count decreased | 1 (3) | 14 (44) |
| Peripheral sensory neuropathy | 1 (3) | 4 (13) |
| Platelet count decreased | 1 (3) | 6 (19) |
| Stomatitis | 1 (3) | 10 (31) |
| White blood cell count decreased | 1 (3) | 10 (31) |
| Alopecia | 0 | 3 (9) |
| Dehydration | 0 | 3 (9) |
| Edema | 0 | 2 (6) |
| Febrile neutropenia | 0 | 2 (6) |
| Hiccups | 0 | 3 (9) |
| Hypokalemia | 0 | 3 (9) |
| Hypomagnesemia | 0 | 4 (13) |
| Hypophosphatemia | 0 | 3 (9) |
| Nausea | 0 | 19 (59) |
| Neutropenia | 0 | 2 (6) |
| Palmar-plantar [erythrodysesthesia](https://www.google.com/search?rlz=1C1GCEU_enUS849US849&q=erythrodysesthesia&spell=1&sa=X&ved=2ahUKEwjxsuzNuqnoAhXGknIEHYC2B58QkeECKAB6BAgOECY) syndrome | 0 | 13 (41) |
| Pulmonary embolism | 0 | 2 (6) |
| Vasculitis | 0 | 2 (6) |
| Weight decreased | 0 | 3 (9) |

*AE* adverse event.

**References**

1. Fuchs CS, Doi T, Jang RW, et al. Safety and efficacy of pembrolizumab monotherapy in patients with previously treated advanced gastric and gastroesophageal junction cancer: phase 2 clinical KEYNOTE-059 trial. JAMA Oncol. 2018;4:e180013.

2. Shitara K, Ozguroglu M, Bang YJ, et al. Pembrolizumab versus paclitaxel for previously treated, advanced gastric or gastro-oesophageal junction cancer (KEYNOTE-061): a randomised, open-label, controlled, phase 3 trial. Lancet. 2018;392:123–33.

3. Shitara K , Van Cutsem E, Bang YJ, et al. Efficacy and safety of pembrolizumab or pembrolizumab plus chemotherapy vs chemotherapy alone for patients with first-line, advanced gastric cancer: the KEYNOTE-062 phase 3 randomized clinical trial. JAMA Oncol. 2020;6:1571–80.

4. Kulangara K, Zhang N, Corigliano E, et al. Clinical utility of the combined positive score for programmed death ligand-1 expression and the approval of pembrolizumab for treatment of gastric cancer. Arch Pathol Lab Med. 2019;143:330–7.
